# Supplementary figures and images for: Comparative Study of Pine Reference Genomes Reveals Transposable Element Interconnected Gene Networks
Source: Genes (Basel). 2020 Oct 16;11(10):1216. doi: 10.3390/genes11101216 (PMC7602945; doi:10.3390/genes11101216)

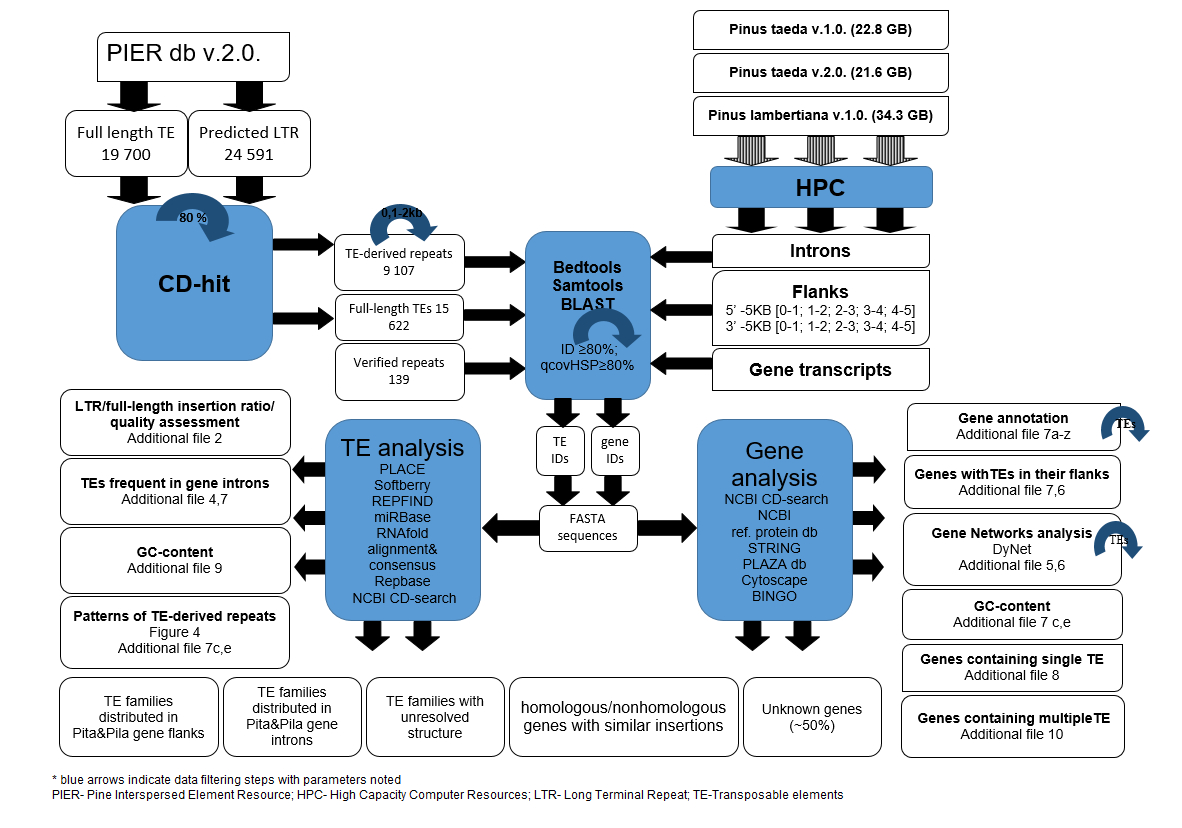

Supplement: Supplementary file 1 [file genes-11-01216-s001.zip › MDPI_genes_893100_15oct20/Add_file_1_modif.jpg]
